# Using qualitative comparative analysis (QCA) in systematic reviews of complex interventions: a worked example

**DOI:** 10.1186/2046-4053-3-67

**Published:** 2014-06-20

**Authors:** James Thomas, Alison O’Mara-Eves, Ginny Brunton

**Affiliations:** 1 EPPI-Centre, Social Science Research Unit, Institute of Education, 18 Woburn Square, London WC1H 0NR, UK

**Keywords:** research synthesis, systematic review, qualitative comparative analysis, QCA, complex interventions, theory, heterogeneity

## Abstract

**Background:**

Systematic reviews that address policy and practice questions in relation to complex interventions frequently need not only to assess the efficacy of a given intervention but to identify which intervention - and which intervention components - might be most effective in particular situations. Here, intervention replication is rare, and commonly used synthesis methods are less useful when the focus of analysis is the identification of those components of an intervention that are critical to its success.

**Methods:**

Having identified initial theories of change in a previous analysis, we explore the potential of qualitative comparative analysis (QCA) to assist with complex syntheses through a worked example. Developed originally in the area of political science and historical sociology, a QCA aims to identify those configurations of participant, intervention and contextual characteristics that may be associated with a given outcome. Analysing studies in these terms facilitates the identification of necessary and sufficient conditions for the outcome to be obtained. Since QCA is predicated on the assumption that multiple pathways might lead to the same outcome and does not assume a linear additive model in terms of changes to a particular condition (that is, it can cope with ‘tipping points’ in complex interventions), it appears not to suffer from some of the limitations of the statistical methods often used in meta-analysis.

**Results:**

The worked example shows how the QCA reveals that our initial theories of change were unable to distinguish between ‘effective’ and ‘highly effective’ interventions. Through the iterative QCA process, other intervention characteristics are identified that better explain the observed results.

**Conclusions:**

QCA is a promising alternative (or adjunct), particularly to the standard fall-back of a ‘narrative synthesis’ when a quantitative synthesis is impossible, and should be considered when reviews are broad and heterogeneity is significant. There are very few examples of its use with systematic review data at present, and further methodological work is needed to establish optimal conditions for its use and to document process, practice, and reporting standards.

## Background

Systematic reviews that address policy and practice questions frequently need to not only assess the efficacy of a given intervention but to identify which intervention, out of a range of possibilities, might be relevant in the particular situation [1]. Network meta-analysis has been receiving widespread interest as it enables a reviewer indirectly to compare the relative effectiveness of Intervention A with Intervention C, even where existing studies separately compare Interventions A and C directly with a third Intervention (B) [2]. However, knowing which intervention worked best in a given context is only part of the problem in many areas of public policy, where complex multi-component interventions are tailored for use in different situations. Here, intervention replication is rare [3], and when the focus of analysis is the identification of those components of an intervention that are critical to its success, network meta-analysis may require more data to operate effectively than are available [4].

Moreover, statistical methods that are based on the exploration and explanation of correlations between variables are sometimes ill-suited in the analysis of causal pathways. Since there may be multiple ‘paths’ that are able to lead to a successful outcome in different contexts, a particular component ‘x’ may be present and critical to the success of intervention variant A, as well as being present in intervention B - which was not successful. But because correlational approaches test simultaneously for the ‘success’ and ‘failure’ of covariates, they are unable to identify the importance of the component ‘x’ in intervention A when it is also present in unsuccessful intervention B. (Testing for interaction is usually impossible in systematic reviews because of a lack of data.) For example, in an analysis that is examining whether the training of intervention providers results in better outcomes, a correlational analysis will require that the training of intervention providers is associated with good outcomes AND that the absence of their training is associated with poorer outcomes. If there are multiple approaches to achieving effectiveness though, it may be that some (or all) of the interventions where training did not occur had good reasons for this (for example, more experienced providers were recruited). This reason, however, would not be picked up in the analysis, and the importance of training in the interventions that did train providers would be lost.

The identification of critical intervention components relates to a common logic discussed in the literature about causation, necessity and sufficiency [5,6], and it is a valuable framework to use when thinking about expressing review findings for potential review users: What intervention components are necessary to put in place in order to ensure success in a given situation? And which components, or combination of components, are sufficient to gain a given outcome in a given situation? If we are interested in identifying necessary and sufficient intervention components to recommend in different situations, what are the analytical techniques that enable us to do this? In particular, we need robust and systematic methods that enable us to compare and contrast differences in intervention strategy and context, and relate these to the outcomes obtained.

The above sets the context for this paper: a need to identify important components of interventions when making commissioning decisions, but a lack of established methods of synthesis which enable such investigations. We therefore examine an analytical technique, ‘qualitative comparative analysis’ (QCA), which has been designed to overcome some of the limitations outlined above. Through a worked example, we demonstrate its application to systematic reviews and examine its utility when synthesising the results of complex interventions. We will discuss its foundational principles in relation to synthesis, but refer readers to primary methodological sources for a more complete account of its logic. (In particular, please see *Configurational Comparative Methods*[7] and *Redesigning Social Inquiry*[8]; software and a user manual developed by Ragin are freely available at http://www.u.arizona.edu/~cragin/fsQCA/.)

### Introduction to qualitative comparative analysis

Developed on an ongoing basis by Charles Ragin and colleagues since the late 1980s, QCA was originally designed to facilitate research in political science and historical sociology. The types of analyses for which it was developed typically involved the comparison of nation states with one another; this is a classic ‘small N-many variables’ scenario, where the number of examples of a phenomenon is small (for example, OECD countries) and the number of variables that might explain a given outcome might be large (for example, the factors which give rise to the creation of generous welfare states) [7]^a^. Reviewers face similar challenges when synthesising evaluations of complex interventions, where there are often a limited number of studies and a large number of possible factors that might explain differences in their findings (for example, participants, interventions, context, outcome measurement, study design, comparator, *etcetera*).

A characteristic that distinguishes QCA from the statistical methods discussed earlier is that it takes a ‘case’ rather than ‘variable’ perspective in its analysis. In so doing, it aims to transcend the qualitative/quantitative divide [9], changing the focus of investigation from the individual study to the different configurations of intervention, participant, and contextual characteristics that together are responsible for the intervention resulting, or not resulting, in the outcome of interest. ‘Simply said, a configuration is a specific combination of factors (or stimuli, causal variables, ingredients, determinants, *etcetera* - we call these *conditions* in CCM [Configurational Comparative Methods] terminology) that produces a given *outcome* of interest’ [10].

As highlighted above, the identification of necessary and sufficient programme characteristics is a potentially extremely useful product of a synthesis that aims to generate policy/practice relevant findings. QCA appears to be a good fit with this framework, since its focus on configurations of factors aims explicitly to identify these necessary and sufficient conditions; indeed, these are the unit of analysis, rather than the individual research study. The use of the term *condition* is important in a QCA analysis, and contrasts to some degree with the term *variable* in the statistical methods discussed above. Ragin distinguishes between the analysis of ‘independent variables’, where factors are necessarily distinct, and the configurational analysis of causes and conditions. In the former, investigations of combinations of conditions are hampered by collinearity (when variables have a linear relationship), whereas the fact that some conditions may be related to one another is expected in a QCA, and is part of the Boolean set logic (intersection) that underpins the analysis [8]. While there are statistical techniques to deal with this (for example, interaction terms), in the context of research synthesis these are rarely possible due to the small number of studies available in the analysis.

**Figure 1:**
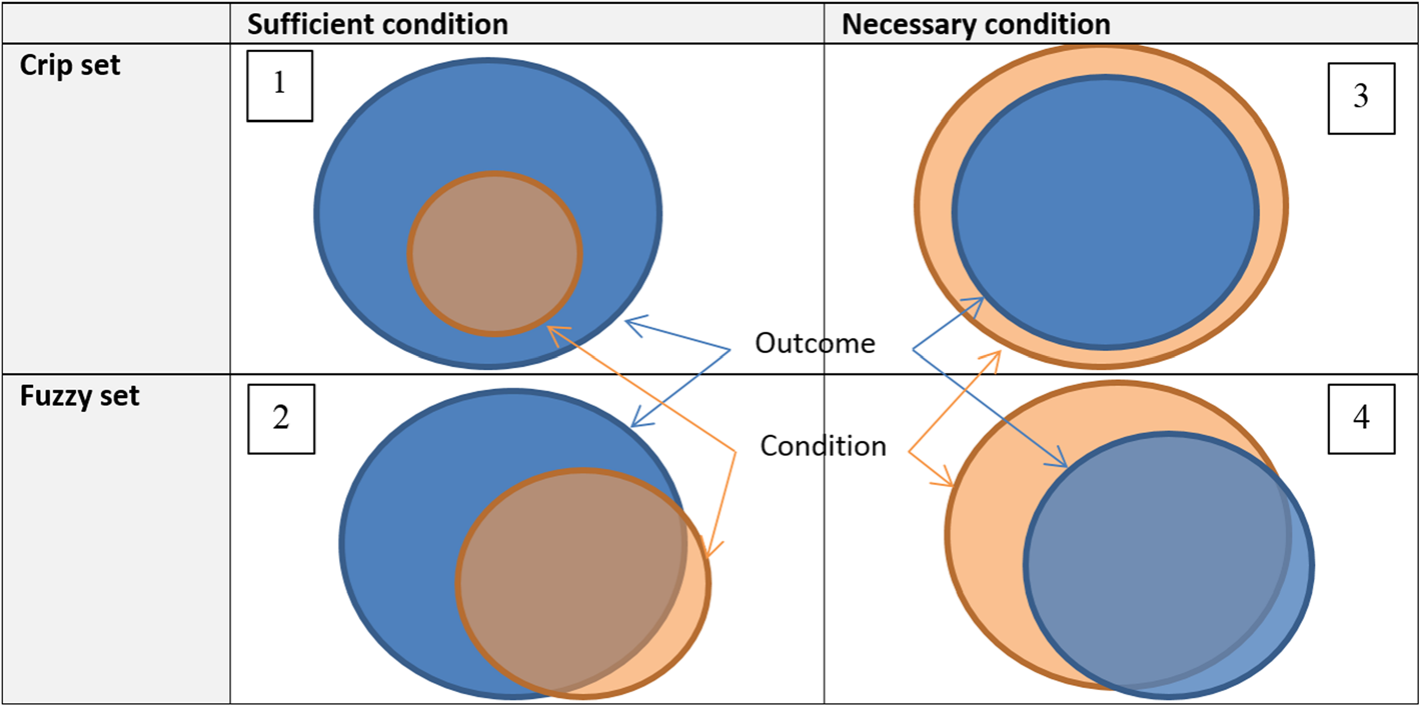
Necessary and sufficient conditions.

The final characteristic of QCA to describe here is that it is an analytical framework based on Boolean set logic. Sets and set relations, Ragin argues, are the basis of almost all social science theory, and it is through the use of set theoretic principles that Ragin seeks to transcend the qualitative/quantitative divide [8]. For example, if all smoking cessation interventions involve the provision of an information leaflet, then those interventions can be considered to be a *subset* of the set of all interventions (on weight loss, CVD, *etcetera*) that provide information leaflets. Thinking in these terms provides set-based analytical algorithms (see below), and critically, combinations of conditions can themselves also be conceptualised in set-theoretic form and analysed in the same way.Figure 1 illustrates necessary and sufficient set relationships in graphical form. Outcomes are denoted in the darker (blue) colour, and conditions in the lighter (peach) colour. Illustration 1 shows a perfect sufficient condition, where all studies with a particular characteristic (or set of characteristics) are associated with the given outcome. Illustration 2 shows a more ‘lifelike’ version of this relationship, in which a proportion of the studies with the characteristic(s) display the outcome, whereas some do not (known as ‘quasi-sufficiency’). In illustrations 1 and 2, it is clear that the condition need not be present in all interventions in which the outcome occurs for it to be considered sufficient, and the proportion of the studies for which this is the case is denoted by the metric ‘coverage’. This metric indicates the extent to which the configuration analysed is the only known path to the outcome; a related metric, ‘consistency’, indicates how often the given configuration occurs. (These metrics are illustrated further in the worked example below.) Illustrations 3 and 4 describe the much rarer situation of a necessary condition. Illustration 3 depicts a ‘perfect’ necessary situation, in which the set of interventions with the given outcome is a subset of all studies with a particular characteristic (or characteristics). Illustration 4 completes the picture, showing a quasi-necessary condition; this is displayed for conceptual completeness, but is not a focus of many analyses due to: a) its similarity to quasi-necessity, and b) the fact that necessity is itself often very difficult to conceptualise and even more difficult to prove, as there may be numerous ways in which the given outcome may be obtained which might be out of the scope of the analysis in question to include.

In ‘fuzzy-set QCA’ (or fsQCA), partial membership in sets are permitted. Thus, rather than an intervention either being a member of the ‘effective’ set of interventions or not, it can also be partially in or out of this set. This permits greater flexibility in categorisation (for example, allowing the relative intensities of interventions to be considered in the analysis). When using fuzzy sets, it is important that the set membership positions (for example, 1 for full membership in a set; 0 for full non-membership; and 0.5 being precisely neither fully in nor fully out of the set) are appropriately scaled (see example below) and are ‘qualitatively anchored’ to the meaning of the outcome in question [8]. For example, rather than simply scaling the effect size estimates from a set of studies to lie between 0 and 1 in a linear fashion, it is important to consider the meaning of an outcome denoting full membership in the set of effective interventions; that is, the clinical/practical significance of the outcome must be considered, and it should by no means be a given that a statistically significant, but clinically insignificant, result be considered an ‘effective’ outcome.

The result of QCA takes the form of a number of configurations of various participant, intervention, and contextual characteristics that are (or are not) present when the intervention has been successful (or not) in obtaining the desired outcome. Analysing studies in these terms facilitates the identification of necessary and sufficient conditions for the outcome to be obtained, a feature of QCA that may facilitate the translation of its findings to practical situations. Since QCA seeks to identify ‘causal recipes, not net effects’ (the idea that multiple pathways might lead to the same outcome), and does not assume a linear additive model in terms of changes to a particular condition (that is, can cope with tipping points), QCA appears not to suffer from some of the limitations of the statistical methods discussed above. In view of this, we present a worked example of QCA where we have synthesised a group of studies evaluating complex interventions. We are interested here in examining QCA’s utility within this sort of synthesis: Does it indeed overcome some of the limitations identified above? And, in turn, what are the limitations of using QCA?

## Methods

### Aims and rationale

The aim of this section of the paper is to demonstrate QCA methods and to examine their utility for synthesising a set of complex interventions. Our data are a subset of studies identified in a review of community engagement in interventions for public health and health promotion [11]: those directed toward expectant and new mothers to promote breastfeeding. The study aimed to explore which methods (and combinations of methods) of community engagement were present in effective interventions. To that end, the following three conditions were selected for testing:

1. Empowerment: studies in which members of the community define the health need.

2. Involvement in intervention design: interventions in which community members were involved (that is, consulted, collaborating, or leading) in the design of the intervention.

3. Lay-led intervention: interventions in which the delivery of the intervention is led by lay persons (including peers, community health workers, *etcetera*).

These conditions were chosen because they represented different aspects of the intensity, source and ‘ownership’ of engagement. Each condition on its own had been shown to have a statistically significant effect on health behaviour outcomes, but it was unknown whether combinations of these aspects were effective [11]. Some have suggested that interventions amongst disadvantaged populations that are completely empowerment-driven will be most effective [12,13], while others have noted that pragmatic interventions that utilise public involvement to improve them can also be successful [14]. It is possible that the results obtained by sub-dividing the studies according to these different conceptualisations of engagement differ from situation to situation and from outcome to outcome, and another analysis might include additional variables. For the purposes of this illustration, however, we will discuss the application of QCA within the three conditions itemised above, since these map directly to our overarching conceptual framework of community engagement. As our analysis will demonstrate, this initial conceptualisation did not enable us to explain observed differences between the studies, and two additional conditions that have greater explanatory value were developed through the synthesis using QCA.

### Data

#### The included studies

The broader project from which this paper’s dataset was drawn was a large, multi-method systematic review of public health and health promotion interventions that incorporated community engagement and were delivered to disadvantaged populations [11]. The original review included 319 studies covering a range of modifiable health issues such as smoking, alcohol abuse, substance abuse, and obesity. In the original report, a subset of 131 of the 319 studies were analysed in a statistical meta-analysis; these 131 studies represented a narrower range of health topics, outcomes, and evaluation designs than in the broader systematic review. Importantly, the meta-analysis indicated substantial statistical heterogeneity amongst the studies, and there was clear conceptual variation across the studies despite the narrower inclusion criteria for the meta-analysis.

For the QCA analyses, where we analyse combinations of conditions contributing to the effectiveness of the interventions, we focus on a subset of studies that aimed to promote breastfeeding and reported a binary outcome measure (n = 12). These studies are a conceptually coherent group with sufficient diversity and similarity to demonstrate a synthesis using QCA effectively.

Please note that this selection of data departs somewhat from the purposive selection of cases that textbooks on QCA describe. The guidance recommends that cases are selected on the basis that they are similar enough to be comparable, but also that a ‘maximum of *heterogeneity* over a minimum number of cases should be achieved’ (original emphasis) [7]. Additionally, researchers should seek cases with both positive and negative outcomes. In the context of a systematic review however, such purposive (or ‘theoretical’) sampling can be difficult to achieve, since the set of studies available may not contain both positive and negative findings relating to the same outcome. The positive way in which heterogeneity is viewed is also notable, since the analysis depends upon differences in order to provide explanations; this is something that is often portrayed as a problem in systematic reviews, where heterogeneity can complicate a meta-analysis by suggesting that all studies are not estimating the same underlying effect.

## Results

### Stages of synthesis using qualitative comparative analysis

According to Rihoux and Ragin (2009) [7], there are six stages in a QCA:

1. building the data table,

2. constructing a ‘truth table’,

3. resolving contradictory configurations,

4. Boolean minimisation,

5. consideration of the ‘logical remainders’ cases, and

6. interpretation.

We will follow the six stages in the following example, and because this is an early worked example of QCA in synthesis, we will retain stage 5: consideration of ‘logical remainders’. However, this stage was not necessary in our example, and further methodological work will be required to ascertain whether this stage can be omitted from most syntheses using QCA or whether it has a useful role to play in certain situations.

#### Stage 1: Building the data table

The data table consists of rows that represent studies, while the columns represent conditions (characteristics of the cases) and the outcome/s. The data table for these analyses is shown in Table 1.

**Table 1:** Data table for breastfeeding interventions that incorporate community engagement

**Study**	**Conditions**					**Outcome**	
	***Empowerment***	***Design***	***Lay-led***	***Quality***	***Intensity***	***Effect size (raw odds ratio)***	***Highly effective intervention fuzzy set***
Anderson (2005) [15]	0	0	1	1	1	8.458	1.000
Caulfield (1998) [16]	0	0	1	0.333	1	3.783	1.000
Chapman (2004) [17]	1	1	0	1	0	1.751	0.666
Grummer-Strawn (1997) [18]	0	0	0	0	0	1.927	0.666
Karanja (2010) [19]	0	1	0	0	0	0.463	0.000
Kistin (1994) [20]	0	0	1	0	0	5.397	1.000
Long (1995) [21]	0	0	1	0	0	1.729	0.333
McInnes (1998) [22]	1	1	1	0	0	1.614	0.333
Pugh (2001) [23]	0	0	0	1	1	6.000	1.000
Pugh (2002) [24]	0	0	0	1	1	2.786	0.666
Schafer (1998) [25]	0	1	1	1	1	8.458	1.000
Shaw (1999) [26]	0	0	1	0	0	2.317	0.666

Membership in the conditions in this dataset is almost exclusively binary: cases are either members (represented by a ‘1’) or non-members (represented by a ‘0’) of a condition. These are also referred to as ‘crisp’ sets. As defined above, the conditions are empowerment, involvement in intervention design, and lay-led intervention*.* Note that there are an additional two conditions, ‘Quality’ and ‘Intensity’, included as columns in Table 1. These will be described later in the paper as they emerged as a part of the iterative QCA process (that is, they were not specified *a priori*).

As mentioned above, transposing ‘purposive’ sampling techniques from primary research can be difficult in the context of a systematic review because we cannot necessarily identify positive and negative cases; we can only use the studies that have evaluated the interventions and outcomes of concern. The sample of studies that we have, therefore, is more akin to an unbiased or ‘population’ sample - in that we have all the studies (that we can find) that evaluate a given intervention. If we have little heterogeneity between results, there is little the QCA can do to help us identify sufficient conditions for success.

The outcome in this dataset is an indicator of the effectiveness of the interventions, which were all evaluated in controlled trials (both randomised and non-randomised). For the non-randomised studies, we have no reason to believe that the participants in one condition or another were more likely to breastfeed before the start of the intervention. The original metric used in the meta-analysis to estimate the magnitude of the outcome was an effect size estimate^b^ that compared the health behaviours of participants in the intervention group to those in the control group at immediate post-test (that is, directly after the intervention finished). In most cases, the measure was the number of mothers breastfeeding at a given time point. Note that all but one of the interventions were effective (in a systematic review, it is not always possible to have clearly differentiated positive and negative cases), so the outcome for the QCA analyses was membership in the set of *highly* effective interventions. The log of the effect size estimates (odds ratios, OR) were calibrated for use in the QCA analyses by converting them into a fuzzy set that allows for degrees of membership^c^. In this review, we used the following effect size calibration rules:

1. Full membership in the set of ‘highly effective interventions’: if logged OR > .7.

2. More in than out of the set: .4 < logged OR ≤ .7.

3. More out than in the set: 0 < logged OR ≤ .4.

4. Fully out of the set: logged OR ≤0.

In Table 1, the original raw effect sizes are shown in the column ‘Effect size (raw odds ratio)’ and the calibrated fuzzy set outcome is shown in the column ‘Highly effective intervention fuzzy set’. (Calibration of fuzzy sets is a complex topic, and for further information we recommend Part II of [8].)

#### Stage 2: Constructing the truth tables

Once the data have been prepared, the focus of analysis moves from individual studies to the different configurations of conditions that are associated with the outcome of interest. As noted above, conditions are characteristics of the cases. Different combinations of conditions are referred to as *configurations*. For *k* conditions, there are 2^*k*^ configurations. In the example in Table 2, three possible conditions can be combined in eight (2^3^) different configurations. Each configuration is itself a set, or group, to which studies can be members or not members; studies with the same configuration are included in a set, while studies with different configurations will have membership in different sets.

**Table 2:** Example of possible configurations of three conditions with their set labels

**Conditions**			**Configuration set label**
**Empowerment**	**Lay-led**	**Consulted on design**	
1	1	1	Empowerment*Lay-led*Design
1	1	0	Empowerment*Lay-led* ~ Design
1	0	1	Empowerment* ~ Lay-led*Design
1	0	0	Empowerment* ~ Lay-led* ~ Design
0	1	0	~Empowerment*Lay-led* ~ Design
0	1	1	~Empowerment*Lay-led*Design
0	0	0	~Empowerment* ~ Lay-led* ~ Design
0	0	1	~Empowerment* ~ Lay-led*Design

*Note*. In columns 1 to 3, ‘1’ indicates the given condition is present while ‘0’ indicates that the condition is absent. In column 4, * indicates ‘and’, ~ indicates non-membership in a condition.

The labelling of sets follows certain conventions. An asterisk * is used to combine conditions (equivalent to ‘And’), and a tilde ~ is used to indicate non-membership in a condition. So, for example, a study with the conditions ‘empowerment model evident, with the intervention led by members of the community, but no community involvement in the intervention design’ would be labelled as Empowerment*Lay-led* ~ Design. The fourth column of Table 2 shows the set labels for the various configurations for those three conditions.

Having constructed a data table as described in ‘Stage 1’ above (that is, with an outcome calibrated to the four fuzzy membership levels and with several binary conditions), we then constructed a truth table. A truth table displays the conditions, configurations, and the number of studies with membership in each configuration set. Unlike the original dataset in Table 1, in which each study is a case, a truth table presents each configuration as a case (as in the example in Table 2).

The truth table summarises how many studies within a set (or configuration) are instances of the outcome. In this example, the outcome of interest is whether the intervention was highly effective, and so the truth table indicates how many of the studies within a configuration are members, or partial members, in the set of highly effective interventions. There are four possible kinds of result for each configuration:

1. Positive cases: All studies within a set are instances of the outcome (that is, all studies in the configuration are effective).

2. Negative cases: No studies within a set are instances of the outcome (that is, no studies in the configuration are effective).

3. Contradictions: Some of the studies are instances of the outcome and some are not (that is, studies in the configuration are mixed in terms of their effectiveness; discussed in ‘Stage 3’ below).

4. Remainders: There are no studies in the dataset with that particular configuration of conditions and outcome (discussed in ‘Stage 5’ below).

Since the number of possible configurations increases exponentially as the number of conditions increases, it does not take the addition of many conditions for the number of possible configurations to exceed the number of studies in the analysis. The potential problem arising from this is known as ‘limited diversity’ [27]; that is, the analysis can simply become a description of each individual study, rather than a synthesis where lessons are drawn from across the included studies. The objective is to conduct an analysis that is sufficiently rich, containing the most salient conditions able to explain differences between study outcomes, but where less important conditions are excluded from the analysis. Some primary QCA analyses have used prior analytical strategies including discriminant analysis, factor analysis and cluster analysis to help inform the selection of conditions (for example, [7,28]), though for this example, no such techniques were employed.

The truth table for the first model, in which the conditions (empowerment, design, lay-led) were examined for ‘highly effective interventions’, is presented in Table 3. The rows are in descending order of consistency, which is the metric used in QCA to express how far the pattern of all the cases is consistent with sufficiency. Consistency is defined as a metric that answers the question ‘To what extent is the statement ‘configuration A is necessary for the outcome’ consistent? Technically, this can be computed as follows: (the number of cases with a [1] value on the configuration AND a [1] outcome value, divided by the total number of cases with a [1] outcome value)’ [7]. Ragin discusses appropriate cut-off levels for consistency, arguing that they should be as close to 1 as possible (though the greater the number of studies in a particular configuration, the less likely this becomes), and that it is difficult to justify drawing conclusions when consistency scores are below 0.75 [29]. For our analyses, we adopted a cut-off for consistency of 0.75 or above.

**Table 3:** Truth table for model 1: community engagement models as the conditions and ‘highly effective intervention’ as the outcome

**Empowerment**	**Design**	**Lay-led**	**Number of studies**	**Membership in the ‘highly effective intervention’ set**	**Raw consistency**
**0**	**1**	**1**	**1**	**1**	**1.000**
0	0	1	5	0	0.666
0	0	0	3	0	0.666
1	1	1	1	0	0.333
1	1	0	1	0	0.333
0	1	0	1	0	0
*1*	*0*	*0*	*0*		
*1*	*0*	*1*	*0*		

*Note*. The one configuration with raw consistency > .75 is shown in bold; configurations with raw consistency < .75 are not in bold; configurations with no cases (that is, ‘remainders’) are italicised.

We can see that only one row (that is, one configuration) has sufficient raw consistency (that is, ≥0.75) to be classified as having full membership in the set of ‘highly effective interventions’. However, that configuration consists of only one study.

The truth table for the second model, in which the conditions (empowerment, design, lay-led) were examined for the negated outcome (that is, ‘not highly effective interventions’), is presented in Table 4. Again, only one row (configuration) has sufficient raw consistency to be classified as having full membership in the set of ‘not effective interventions’, but this only represents one study.

**Table 4:** Truth table for model 2: community engagement models as the conditions and ‘not effective interventions’ as the negated set outcome

**Empowerment**	**Design**	**Lay-led**	**Number of studies**	**Membership in the ‘not highly effective intervention’ set**	**Raw consistency**
**0**	**1**	**0**	**1**	**1**	**1**
**1**	**1**	**1**	**1**	**0**	**0.667**
**1**	**1**	**0**	**1**	**0**	**0.667**
**0**	**0**	**0**	**3**	**0**	**0.334**
**0**	**0**	**1**	**5**	**0**	**0.334**
**0**	**1**	**1**	**1**	**0**	**0**
*1*	*0*	*0*	*0*		
*1*	*0*	*1*	*0*		

*Note*. Configurations with raw consistency < .75 are shown in bold; Configurations with no cases (that is, ‘remainders’) are italicised.

We mentioned earlier that it is easier to identify sufficient conditions for an outcome than it is to find necessary conditions because logically there may be (many) other ways of arriving at a given outcome, even if those methods are not present in any of the studies in the analysis. Bearing in mind the above mentioned problems relating to sampling - that we can do no more in a systematic review than find the studies that have already been conducted and cannot collect additional data to fill in gaps, as we might in primary research - we think it unlikely that reviewers will want to identify necessary conditions for complex interventions to generate an outcome. This is because our conceptualisation of complexity requires that we view each intervention and context as potentially unique; therefore, a condition that may be necessary in all the studies we can see may not be necessary in all possible situations.

##### Stage 2B: Checking the quality of the truth table

There are a number of items to check once the truth table has been created in order to ensure that it will perform adequately in the proceeding stages of analysis. Principally, this involves checking that there is a good spread of studies across the different configurations available, and that both positive and negative occurrences of the outcome are well covered. In Tables 3 and 4, we can see that we do not have a reasonable spread in terms of outcomes and data for all but two of the eight possible configurations. Also, in both models, there are two configurations for which there are no cases; these are known as ‘remainders’. Moreover, only one study in each model is ‘consistent’ enough (that is, raw consistency ≥0.75) to proceed to the next stage of analysis.

If the initial check of the truth table reveals areas of concern, for example, a lack of variation among conditions, which might render explanation of the outcome difficult, it is recommended that reviewers return to the conceptual framework that their review is based upon and consider again the dimensions upon which included studies might differ. This, in turn, will prompt a re-examination of the conditions to be used in the synthesis and possibly lead to the incorporation of new, or different, conditions. (See also the suggestions below on resolving contradictory configurations.) Another way of approaching this might be to become more acquainted with the studies themselves, in the expectation that new lines of enquiry will emerge. Whether the former (a more deductive approach) or the latter (inductive) is used, there should be a ‘dialogue’ between the truth table and the studies and concepts it is based on. Some iteration is expected before the final table emerges. (Though discussion of this is outside the scope of this paper, the investigative model adopted by QCA might best be described as ‘abductive’ [30]).

We considered the conditions tested in the initial analysis to be uninformative other than telling us that there are too few studies that have employed an empowerment approach for us to come to a conclusion regarding the efficacy of this method of engagement. We therefore considered whether any other conditions might meaningfully distinguish between highly effective interventions and those interventions with smaller benefits. Based on our understanding of the studies, we decided that two conditions were likely to have a large impact on the effectiveness of the breastfeeding interventions: the intensity of the intervention and the quality of the intervention.

We returned to the 12 original studies and extracted additional information about intervention intensity and quality. Through an iterative process of interacting with the studies and discussion amongst the team, we developed definitions for these two additional concepts. ‘Intensity’ is based on our understanding of the studies’ theories of change, where the most critical period for supporting breastfeeding is immediately pre- and post-partum. Studies in the set of ‘intense’ interventions would recognise this by having frequent contact in this early period, with less intensive support later on. The second condition, ‘quality’, follows some of the principles of engagement identified elsewhere in our review. ‘High quality’ interventions were defined as those that were customisable to women's needs; had multiple support options; emphasised personal contact (for example, face-to-face as opposed to support via telephone or letters); included counselling (tailored information); were delivered in a location that suited the women; and had trained staff (including practice observation). In this example, these attributes have been combined into a single ‘quality’ intervention characteristic. It would be possible to have a separate condition for each quality attribute, though one might quickly run into the problems of ‘limited diversity’ identified earlier (please see discussion on ‘compound’ conditions).

We then reran the analyses with these conditions in the model. The truth table for this model can be seen in Table 5. We can see that three of the four possible configurations, which represent six of the 12 studies, have sufficiently high raw consistency (≥0.75) to indicate membership in the set of highly effective interventions. Having refined our understanding of what makes a highly effective intervention and having identified some of the characteristics that distinguish them from less effective interventions, we then moved on to the next stage of the analysis.

**Table 5:** Truth table for model 3: intervention intensity and quality as the conditions, and ‘highly effective interventions’ as the outcome

**Intensity**	**Quality**	**Number of studies**	**Membership in the ‘highly effective intervention’ set**	**Raw consistency**
**1**	**0**	**1**	**1**	**1.000**
**1**	**1**	**4**	**1**	**0.923**
**0**	**1**	**1**	**1**	**0.750**
*0*	*0*	*6*	*0*	*0.389*

*Note*. Configurations with no cases (that is, ‘remainders’) are italicised and not bolded.

#### Stage 3: Resolving contradictory configurations

Contradictory configurations are sets of studies in which identical configurations of conditions lead to different outcomes. These need to be resolved before the study can proceed as, by definition, they contradict one another in the truth table. They can be identified in crisp sets as consistency values that are non-uniform (that is, values other than 0 or 1). However, checking for contradictions in the context of a fuzzy set outcome is less straightforward than in a crisp set scenario, as studies can be partially in or out of the outcome set. Boolean algorithms help here when data sets are large, and their results can be displayed in the final columns of the truth tables.

In our dataset, we checked for contradictions by referring back to the original data table presented in Table 1. Of the four studies with the potentially contradictory configuration (as indicated by a non-uniform raw consistency) of intensity = 1 and quality = 1, three of the studies were full members of the outcome, while the fourth was ‘more in than out’ (as indicated by the outcome calibration of 0.666). As such, the studies do not contradict each other - they are all highly effective interventions, although the strength of their membership in the outcome set varies slightly. The other potential contradictory configuration of intensity = 0 and quality = 1 only consists of one study, indicating that the raw consistency is non-uniform because of its fuzzy membership in the outcome set, rather than contradiction with another study. We therefore do not have any contradictory configurations.

If contradictory configurations are evident, there are a number of steps that we can take to resolve them: 1) add one or more conditions to the table; 2) remove existing conditions and replace them with others; 3) re-examine the allocation of studies to particular conditions - including outcome - to ensure consistency in interpretation; 4) consider whether variation might be expected given the conceptualisation and operationalization of the outcome; 5) undertake more ‘qualitative’ analysis of the studies to see whether explanatory differences emerge; 6) consider whether the dataset is too heterogeneous; 7) recode contradictory configurations as ‘0’ in the outcome field - presenting them as ‘unclear’; or 8) undertake a ‘vote counting’ procedure, in which the configuration with the most ‘votes’ (studies) is the one on which conclusions are drawn. For further information on these techniques see Rihoux and Ragin (2009) [7]. The decision we took in the worked example was to accept that the theories of change we had begun the analysis with did not distinguish between successful interventions (possibly because our interventions did not cover the full range of theories adequately); we therefore chose option 2, and replaced these non-distinguishing conditions for others which were able to discriminate between those interventions with highly successful outcomes and those which were less successful.

#### Stage 4: Boolean minimisation

At this stage of the analysis, the QCA software (for example, fsQCA [29] or TOSMANA [31]) utilises Boolean minimisation algorithms to analyse the truth table and identify the most logically simple expression of a Boolean formula. Since the purpose of the exercise is synthesis - to draw conclusions across studies - we would like to find solutions which encompass as many of our studies as possible.

Using the consistency threshold of 0.75 in our truth table for model 3 (Table 5), we are left with three rows to enter our analysis. According to these three rows, membership in the effective intervention set can be written as:

intensity*~quality+

intensity*quality+

$$\sim \mathrm{intensity}*\mathrm{quality}\;>\;\mathrm{Outcome}\;\left(\mathrm{highly}\;\mathrm{effective}\;\mathrm{intervention}\right)$$

The Boolean minimisation algorithm will reduce the ‘solution’ of the truth table by identifying the fact that rows 1 and 2 differ only in terms of the presence of the ‘quality’ condition, while rows 2 and 3 only differ in terms of the presence of the ‘intensity’ condition. Since the outcome is ‘highly effective’ as long as either intensity or quality are present, regardless of whether both are present or not, the minimisation routine removes it from the solution, as illustrated in Table 6. Thus, the minimised solution can be written as ‘intensity*quality > Outcome’, where * indicates ‘Or’ (in plain English, this solution would read as ‘the presence of high intensity or high quality are sufficient for the outcome to occur’). The solution coverage of 0.714 indicates the proportion of studies with a highly effective intervention that have either of the two configurations, while the solution consistency of 0.833 gives the proportion of studies with either configuration that obtains the outcome of a highly effective intervention.

**Table 6:** Solution for model 3 with ‘highly effective interventions’ as the outcome

	**Unique coverage**	**Solution coverage**	**Solution consistency**
Intensity	.667	.714	.833
Quality	.619		

#### Stage 5: Consideration of the ‘logical remainders’ cases

A configuration without any cases is known as a ‘remainder’, and the presence of remainders is known as ‘limited diversity’. Model 3 had no rows without any cases and therefore had no remainders, and limited diversity is not a problem for that model.

In other models, particularly those with a greater number of conditions, remainders are likely. When remainders are evident, the analyst is required to consider logical explanations - - and possibly even impute values for the unobserved cases. Any imputation of values should be driven by theory and substantive knowledge. See Schneider and Wagemann 2012 for a discussion of dealing with remainders [32].

#### Stage 6: Interpretation

Once the simplified solution has been identified, the final stage is to interpret the solution in the light of the studies they are based on, the review’s research questions, and the conceptual framework which guides the review. In our example, we find that we have good evidence for concluding that there are two main routes to a highly effective intervention: first, through an intervention where the intervention is high in intensity and second, when the intervention is high quality. A QCA synthesis may stop at this point, or it may go on to develop theory to explain its findings and to increase generalizable messages.

In the context of our original review, the QCA both challenges our starting assumptions and adds nuance. In terms of our overall conceptualisation of community engagement, this analysis suggests that our broad theories of change cannot explain why some interventions in this sub-set of studies obtained better results than others: it appears to be more important that women receive substantial support in the critical period pre- and post-partum. Thus, while our overall report showed that the theories of change examined to begin with are able to differentiate between interventions at a higher level of abstraction [11], making finer-grained distinctions between the relative successes of outcomes from similar interventions requires a focus on other intervention characteristics beyond the type of community engagement utilised.

## Discussion

### Summary of ‘findings’

Our example has demonstrated the use of QCA to synthesise studies in a systematic review. We used a coherent sub-set of studies (evaluations of interventions to promote breastfeeding) from a broader review.

We found through the truth table for models 1 and 2 that different approaches to community engagement did not tell us anything meaningful about what conditions need to be present for a highly effective breastfeeding intervention. By looking at the number of studies with each type of community engagement condition, we can see that the chief problem is a lack of diversity; in particular, only two studies in this dataset used an empowerment model. With so few studies in each configuration, we were unable to gain a consistent picture of necessary and sufficient conditions for achieving a highly effective intervention, with very low solution coverage as a result.

Although our original premises were around community engagement, the preliminary analyses were not in vain. The findings from our initial models tell us that we need more evaluations of breastfeeding interventions that employ different types of community engagement approaches, and that other intervention characteristics must be examined in addition to these approaches. Furthermore, through the iterative ‘dialogue’ between data and truth table -- an important aspect of the ‘abductive’ QCA process -- we were able to identify two conditions that are meaningful: intervention intensity and intervention quality. These two conditions were not in our original scope, but could be useful for developing guidance for practitioners. Although the substantive topic is not the focus of this paper, there are clear recommendations that could be made around the timing and delivery mode of the interventions that are more likely to be associated with highly effective interventions.

### Single and ‘compound’ conditions

When QCA is used in a systematic review, the use of ‘compound’ conditions may be required more often than in primary research, since we are limited to the information available in publications (and cannot ‘observe’ more about each case, which may be possible in primary research). This means that a balance will need to be struck between parsimony - the simplest possible ‘solution’ and complexity - looking at the fine detail as to how each case may differ from another. Our conditions named ‘intensity’ and ‘quality’ were ‘compound’ conditions to differing degrees: both might be understood to be made up of multiple intervention characteristics. High-quality interventions were those, which in line with some of the principles of engagement we had previously identified, appeared to have high-quality interactions between intervention deliverer and participant. These could be operationalised in different ways, such as an emphasis on personal contact, multiple support options, tailoring to individuals, and delivery in a location that suited women. While the ‘intensity’ condition might appear more homogenous, the details of how ‘intensity’ was operationalised across the interventions differed subtly from one another (for example, in terms of who was delivering the intervention, where it was delivered, and precisely how frequently). We could have broken down each condition further in order to represent this heterogeneity in detail, but we would simply have ended up with a list of different interventions. Instead, we chose to ‘drive’ our operationalization of conditions according to theory: ‘quality’ was about how well attuned the intervention was to the way that each participant wanted to be ‘engaged’, and ‘intensity’ was about understanding that it was important to institute breastfeeding promptly after birth before other routines had become established. Focusing on the theory, the ‘why’ a given issue might affect the outcome is a logical way of creating ‘compound’ conditions, since the conditions grouped here will logically co-occur and there may be little to be gained in separating them. A balance needs to be struck, however, between the use of compound conditions to reduce the likelihood of running into problems of limited diversity and the use of conditions that are fine-grained enough to identify causally important differences between interventions.

### Qualitative comparative analysis compared with more established synthesis methods

Unlike a meta-analysis, the focus of this example analysis is very firmly on the configurations of conditions - not on the magnitudes of effect size estimates. This is likely to have both positive and negative implications. On the positive side, any attention given to small, but non-significant, differences in findings between studies is removed, and we instead focus on the configurations that might underpin them (see below). It also prevents any focus on the statistical significance of individual studies. At first, the lack of any visual clues as to the relative magnitude of effect sizes (for example, a forest plot) gives the impression that important information has been hidden. This information is certainly not lost because, in using fuzzy sets, we have incorporated most of these data into the analysis. On the negative side, the move away from individual study effects shows how important the initial decisions regarding fuzzy set calibration are and how sensitive the analysis is likely to be to these decisions. It may be that methods development is required here in order to establish whether and how to perform sensitivity analyses around calibration decisions. For instance, we conducted sensitivity analyses in relation to one study (Pugh 2002 [24]) that could be classified as ‘more in than out’ or ‘more out than in’ depending on how we calibrated the outcome because it was difficult to determine where that particular intervention’s results lay on the ‘moderately effective to highly effective’ continuum. We did find that the calibration made a difference to the analyses - both solution coverage and consistency were reduced in the alternative model, although the main message that the presence of either high intensity or high quality approaches will result in a highly effective intervention was maintained (results of the alternative model are not presented here).

The process of conducting a QCA follows formal steps that are clearly replicable. Claims as to the replicability of the entire analysis are less clear, however, as decisions need to be made by reviewers in the ‘dialogue’ between truth table and the written reports of the studies it is based on. This ‘dialogue’ is something that is contrary to established systematic review methods, since it explicitly encourages post-hoc exploration of study differences, which is driven by knowledge of actual study findings. This reviewer interpretation may be more open to bias and be less replicable than a typical sub-group analysis that aims not to deviate from *a priori* data extraction categories and sub-group divisions. (See below, however, for a discussion of the type of knowledge generated.) While it is possible for this process to be written up in a transparent way that will make clear the decisions made by reviewers, further work is needed in this area to establish reporting standards, striking a balance between accountability and brevity.

### Should qualitative comparative analysis be used to synthesise studies in systematic reviews?

Our example above is one of the very few cases that we know of where QCA has been used with data from a systematic review (others include [33-35]). While there are few examples of QCA being used to synthesise data in systematic reviews, we do think the method has promise, though further work is needed to establish when and how it might be most appropriate. Our interest in the method stemmed from work we were conducting where existing methods seemed to be inadequate. Thus, in systematic reviews of relatively homogenous and simple interventions, existing meta-analytic methods appear to be appropriate and capable tools. When dealing with complex interventions that differ from one another in subtle (and not so subtle) ways, existing methods appear to lack the analytical purchase necessary to generate actionable findings, to identify the causal pathways, and to cope with the lack of replication that is typical in the evaluation literature of such interventions. It appears that QCA may be a useful tool to use when existing statistical methods fall short.

Thus, QCA may be most useful when an effectiveness review finds that a statistical analysis is not viable and a ‘narrative’ synthesis is attempted. This is likely to happen in systematic reviews of complex interventions where accepted practice varies widely and often amounts to a summary of individual study findings under broad headings of intervention type [36]. Reviews of this sort sometimes struggle to conduct a ‘synthesis’ in any meaningful way and become a list of study findings, and can be dangerously over-reliant on statistical significance (or invite the reader to take special note of studies with a statistically significant finding). QCA in these circumstances might offer a more formal structure than current practices and facilitate critical engagement with what we *do* know about in the studies, allowing us to use this to generate theory about how different conditions might interact to produce effect.

We emphasise that, in some cases, it might be fruitful to conduct both a statistical meta-analysis and a QCA, as they address different aspects of questions around effectiveness (discussed in the next section).

### The type of knowledge generated

A full discussion of the ontological and epistemological issues raised in a comparison of the statistical and QCA-oriented analyses discussed here is outside the scope of this paper; however, some general observations are possible.

Methods for statistical meta-analysis and regression are based on those commonly employed in primary epidemiological research, and are based on sampling theory and properties of the normal distribution. Within this way of knowing, as long as the systematic review is conducted in an unbiased way (and is itself not affected by exterior biases, such as publication bias), we have precise metrics for estimating how confident we should be about the precision of our conclusions, and the likely probability that the results seen would be replicated elsewhere. Generalisability is based on the probability that a given outcome is likely in a given population, and further research is recommended when data are insufficient. Within a QCA frame of knowledge, the aim is to identify configurations of conditions, and in so-doing, generate theory which might explain differences in findings between the observed studies. These configurations and the theory they generate are the basis of generalisation to practice and recommendations for research.

It is worth noting that analysing complex interventions in heterogeneous datasets usually requires sub-group analysis using statistical methods. Standard guidance on this, and views on the type of knowledge generated, varies between the very cautious ‘subgroups kill people’ and the pragmatic ‘…and lack of subgroup analysis kills people’ (that is, subgroups may differ from one another due to chance and so be misleading; but subgroup analysis may be the only way to answer some research questions, such as the impact of social class) [37]. Both may be correct in different circumstances, but it is impossible to know which pertains in any given review. QCA comes from a quite different viewpoint in the philosophy of science: while formal statistical subgroup analysis is based on deductive reasoning and the expected warrants for making causal claims; QCA can best be thought of an ‘abductive’ approach, which aims to provide an ‘inference to the best explanation’ based on the available evidence [38]. Despite the challenges inherent in making causal inferences, decision-makers do require the types of knowledge that, at times, can only be gained from sub-group analyses; thus, in rejecting all such analyses, one may risk missing important knowledge. A QCA analysis too may suffer from the same biases and limitations as a statistical sub-group analysis, since few intervention replications are available and individual study results may be idiosyncratic and atypical. However, where useful knowledge cannot be gained through statistical synthesis, the QCA approach offers a much more formal, powerful and considered way of unpicking a complex evidence base than a simple list of individual study findings; while different, the knowledge claims made by this type of analysis might be considered as being similar to those from a standard sub-group analysis in terms of potential bias and potentially higher in terms of their utility and because they aim to provide explanations based on all available evidence, rather than only part of it (as is the case in a traditional sub-group analysis).

### Qualitative comparative analysis and realist reviews

It has been suggested elsewhere that QCA is a good method to use in a realist review [39,40]. A realist review begins with a mechanism, and as shown above, QCA with case selection. QCA might then be able to assist with the identification of patterns and uniformities (that is, ‘mechanisms’) across the data (causal ‘regularities’ and ‘demi-regularities’ [41,42]). Indeed, Ragin and colleagues suggest that ‘Explicit connections give a formal shape to observed regularities that occur in the data set, and this allows for further investigations, as they are dissected to elaborate an ‘explanation’ - an attempt to describe the mechanism at work’ [7]. Thus, QCA might be a way of identifying and understanding the differences between different mechanisms, rather than the later phase of synthesising Context, Mechanism, Outcome (CMO) configurations, and in doing this it may perform a valuable task within a realist review.

## Conclusions

In order to inform policy and practice, decision-makers need to be able to identify the most promising interventions as well as the ‘active ingredients’ within complex interventions. Current statistical methods of synthesis operate well for homogenous datasets, but poorly where there are few replications and where interventions are complex. QCA is a promising method that should be considered when quantitative synthesis cannot explain the between-study heterogeneity observed; in these situations, it might usefully replace the standard fall-back of a narrative synthesis and suggest ways in which particular combinations of intervention characteristics might be associated with improved outcomes. There are very few examples of its use with systematic review data at present, and further methodological work is needed to establish optimal conditions for its use and to document process, practice, and reporting standards.

## Endnotes

^a^More recently, the approach has also been successfully utilised in a range of medium- and large-N scenarios. 8. Cooper B, Glaesser J, Gomm R, Hammersley M: *Challenging the qualitative - quantitative divide.* London: Bloomsbury Publishing; 2012.

^b^Effect size estimates for participants were calculated using standard techniques (10), adjusting for cluster allocation (11) where necessary.

^c^NB. The outcome could also have been operationalised as a crisp set, with interventions being coded as, for example, either highly effective or not highly effective. We opted for a fuzzy set to better capture the diversity of the intervention effectiveness and to reflect uncertainty about the real-world impact of effect sizes of different magnitudes. Please see Ragin [7] for further information on fuzzy set calibration.

## Abbreviations

CVD: cardiovascular disease; fsQCA: fuzzy-set qualitative comparative analysis; OECD: Organisation for Economic Co-operation and Development; OR: odds ratio; QCA: qualitative comparative analysis.

## Competing interests

The authors declare that they have no competing interests.

## Authors’ contributions

All authors contributed equally to the paper. Writing was led by JT, the QCA analyses were ‘run’ by AOM, and GB contributed topic as well as methodological expertise. All authors read and approved the final manuscript.

## References

[B1] Noyes J Gough D Lewin S Mayhew A Michie S Pantoja T Petticrew M Pottie K Rehfuess E Shemilt I Shepperd S Sowden A Tugwell P Welch V A research and development agenda for systematic reviews that ask complex questions about complex interventions J Clin Epidemiol 2013 65 1262 1270 23953084 10.1016/j.jclinepi.2013.07.003

[B2] Salanti G Schmid C Research Synthesis Methods: Special Issue On Network Meta-Analysis 2012 Chichester: John Wiley & Sons 10.1002/jrsm.1050 26062081

[B3] Milat A Bauman A Redman S Curac N Public health research outputs from efficacy to dissemination: a bibliometric analysis BMC Public Health 2011 11 934 22168312 10.1186/1471-2458-11-934 PMC3297537

[B4] Grant ES Calderbank-Batista T Network Meta-Analysis for Complex Social Interventions: Problems and Potential J Soc Soc Work Res 2013 4 4 406 420

[B5] Parascandola M Weed DL Causation in epidemiology J Epidemiol Community Health 2001 55 905 912 11707485 10.1136/jech.55.12.905 PMC1731812

[B6] Phillips C Goodman K Causal criteria and counterfactuals; nothing more (or less) than scientific common sense Emerg Themes Epidemiol 2006 3 5 16725053 10.1186/1742-7622-3-5 PMC1488839

[B7] Rihoux B Ragin C Configurational Comparative Methods: Qualitative Comparative Analysis (QCA) And Related Techniques 2009 London: Sage

[B8] Ragin C Redesigning Social Inquiry: Fuzzy Sets And Beyond 2008 London: University of Chicago Press

[B9] Cooper B Glaesser J Gomm R Hammersley M Challenging The Qualitative-Quantitative Divide 2012 London: Bloomsbury Publishing

[B10] Rihoux B Ragin C Rihoux B, Ragin C Why Compare? Why configurational comparative methods? Configurational Comparative Methods 2009 London: Sage xvii xx

[B11] O’Mara-Eves A Brunton G McDaid D Oliver S Kavanagh J Jamal F Matosevic T Harden A Thomas J Community engagement to reduce inequalities in health: a systematic review, meta-analysis and economic analysis Public Health Res 2013 1 4 25642563

[B12] Yoder J Kahn A Toward a feminist understanding of women and power Psychol Women Q 1992 16 381 388

[B13] Hicks L Feminist analysis of empowerment and community in Art education Stud Art Educ 1990 32 36 46

[B14] Cornwall A Democratising Engagement 2008 London: Demos

[B15] Anderson AK Damio G Young S Chapman DJ Perez-Escamilla R A randomized trial assessing the efficacy of peer counseling on exclusive breastfeeding in a predominantly Latina low-income community Arch Pediatr Adolesc Med 2005 159 9 836 41 16143742 10.1001/archpedi.159.9.836

[B16] Caulfield LE Gross SM Bentley ME Bronner Y Kessler L Jensen J Weathers B Paige DM WIC-based interventions to promote breastfeeding among African-American Women in Baltimore: effects on breastfeeding initiation and continuation J Hum Lact 1998 14 1 15 22 9543954 10.1177/089033449801400110

[B17] Chapman DJ Damio G Young S Perez-Escamilla R Effectiveness of breastfeeding peer counseling in a low-income, predominantly Latina population: a randomized controlled trial Arch Pediatr Adolesc Med 2004 158 9 897 902 15351756 10.1001/archpedi.158.9.897

[B18] Grummer-Strawn LM Rice SP Dugas K Clark LD Benton-Davis S An evaluation of breastfeeding promotion through peer counseling in Mississippi WIC clinics Matern Child Health J 1997 1 1 35 42 10728224 10.1023/a:1026224402712

[B19] Karanja N Lutz T Ritenbaugh C Maupome G Jones J Becker T Aickin M The TOTS community intervention to prevent overweight in American Indian toddlers beginning at birth: A feasibility and efficacy study J Community Health 2010 35 6 667 675 20508978 10.1007/s10900-010-9270-5 PMC4058573

[B20] Kistin N Abramson R Dublin P Effect of peer counselors on breastfeeding initiation, exclusivity, and duration among low-income urban women J Hum Lact 1994 10 1 11 15 7619241 10.1177/089033449401000121

[B21] Long DG Funk-Archuleta MA Geiger CJ Mozar AJ Heins JN Peer counselor program increases breastfeeding rates in Utah Native American WIC population J Hum Lact 1995 11 4 279 284 8634104 10.1177/089033449501100414

[B22] McInnes RJ The Glasgow Infant Feeding Action Research Project: an evaluation of a community based intervention designed to increase the prevalence of breastfeeding in a socially disadvantaged urban area [Summary] 1998 Paediatric Epidemiology and CommunityHealth (PEACH) Unit, Department of ChildHealth, University of Glasgow

[B23] Pugh Linda C Milligan Renee A Brown Linda P The Breastfeeding Support Team for low-income, predominantly-minority women: a pilot intervention study Health Care Women Int 2001 22 5 501 515 11508101 10.1080/073993301317094317

[B24] Pugh LC Milligan RA Frick KD Spatz D Bronner Y Breastfeeding duration, costs, and benefits of a support program for low-income breastfeeding women Birth 2002 29 2 95 100 12000411 10.1046/j.1523-536x.2002.00169.x

[B25] Schafer E Vogel MK Viegas S Hausafus C Volunteer peer counselors increase breastfeeding duration among rural low-income women Birth 1998 25 2 101 6 9668744 10.1046/j.1523-536x.1998.00101.x

[B26] Shaw E Kaczorowski J The effect of a peer counseling program on breastfeeding initiation and longevity in a low-income rural population J Hum Lact 1999 15 1 19 25 10578771 10.1177/089033449901500108

[B27] Cooper B Glaesser J Qualitative Work And The Testing And Development Of Theory: Lessons From A Study Combining Cross-Case And Within-Case Analysis Via Ragin’s QCA Forum: Qual Soc Res 2012 13 2

[B28] Berg-Schlosser D Cronqvist L Macro-quantitative vs. macro-qualitative methods in the social sciences: an example from empirical democratic theory employing new software Hist Soc Res 2005 30 154 175

[B29] Fuzzy-Set/Qualitative Comparative Analysis 2.0 http://www.u.arizona.edu/~cragin/fsQCA/citing.shtml

[B30] Rapezzi C Ferrari R Branzi A White coats and fingerprints: diagnostic reasoning in medicine and investigative methods of fictional detectives BMJ 2005 331 1491 1494 16373725 10.1136/bmj.331.7531.1491 PMC1322237

[B31] Cronqvist L Tosmana: Tool for Small-N Analysis [Computer Programme], Version 1.3.2.0 2011 Trier: University of Trier

[B32] Schneider C Wagemann C Set-theoretic methods for the social sciences: a guide to Qualitative Comparative Analysis 2012 Cambridge University Press

[B33] Candy B King M Jones L Oliver S Using qualitative synthesis to explore heterogeneity of complex interventions BMC Med Res Methodol 2011 11 124 21871083 10.1186/1471-2288-11-124 PMC3178541

[B34] Candy B King M Jones L Oliver S Using qualitative evidence on patients’ views to help understand variation in effectiveness of complex interventions: a qualitative comparative analysis Trials 2013 14 179 23777465 10.1186/1745-6215-14-179 PMC3693880

[B35] Pratchett L Durose C Lowndes V Smith G Stoker G Wales C Empowering communities to influence local decision making: systematic review of the evidence 2009 London: Department for Communities and Local Government

[B36] Thomas J Harden A Newman M Gough D, Oliver S, Thomas J Synthesis: combining results systematically and appropriately An Introduction to Systematic Reviews 2012 London: Sage 179 226

[B37] Petticrew M Tugwell P Kristjansson E Oliver S Ueffing E Welch V Damned if you do, damned if you don’t: subgroup analysis and equity J Epidemiol Community Health 2012 66 95 98 21652518 10.1136/jech.2010.121095

[B38] Lipton P Inference to the Best Explanation 2004 2 London: Routledge

[B39] Sager F Andereggen C Dealing with complex causality in realist synthesis: the promise of Qualitative Comparative Analysis Am J Eval 2012 33 60 78

[B40] Befani B Ledermann S Sager F Realistic evaluation and QCA: conceptual parallels and an empirical application Evaluation 2007 13 171 192

[B41] Causality for Beginners http://eprints.ncrm.ac.uk/245/

[B42] Shepperd S Lewin S Straus S Clarke M Eccles MP Fitzpatrick R Wong G Sheikh A Can we systematically review studies that evaluate complex interventions? PLoS Med 2009 6 e1000086 19668360 10.1371/journal.pmed.1000086 PMC2717209

